# The Madrid Affective Database for Spanish (MADS): Ratings of Dominance, Familiarity, Subjective Age of Acquisition and Sensory Experience

**DOI:** 10.1371/journal.pone.0155866

**Published:** 2016-05-26

**Authors:** José A. Hinojosa, Irene Rincón-Pérez, Mª Verónica Romero-Ferreiro, Natalia Martínez-García, Cristina Villalba-García, Pedro R. Montoro, Miguel A. Pozo

**Affiliations:** 1 CAI Cartografia Cerebral, Universidad Complutense, Madrid, Spain; 2 Facultad de Psicología, Universidad Complutense, Madrid, Spain; 3 Instituto Pluridisciplinar, Universidad Complutense, Madrid, Spain; 4 Department of Psychology, Lancaster University, Lancaster, United Kingdom; 5 Departamento de Psicología Básica I, UNED, Madrid, Spain; University of Florida, UNITED STATES

## Abstract

The current study presents ratings by 540 Spanish native speakers for dominance, familiarity, subjective age of acquisition (AoA), and sensory experience (SER) for the 875 Spanish words included in the Madrid Affective Database for Spanish (MADS). The norms can be downloaded as supplementary materials for this manuscript from https://figshare.com/s/8e7b445b729527262c88 These ratings may be of potential relevance to researches who are interested in characterizing the interplay between language and emotion. Additionally, with the aim of investigating how the affective features interact with the lexicosemantic properties of words, we performed correlational analyses between norms for familiarity, subjective AoA and SER, and scores for those affective variables which are currently included in the MADs. A distinct pattern of significant correlations with affective features was found for different lexicosemantic variables. These results show that familiarity, subjective AoA and SERs may have independent effects on the processing of emotional words. They also suggest that these psycholinguistic variables should be fully considered when formulating theoretical approaches to the processing of affective language.

## Introduction

Most studies exploring the interdependence between language and emotion have been based on the assumptions of dimensional models that conceptualize emotions along three dimensions [1, 2]. The valence dimension represents the polarity of the emotion (ranging from pleasant to unpleasant), whereas the dimension of arousal reflects its intensity (ranging from calming to exciting). A third dimension, dominance, refers to the degree of control experienced by an individual over a stimulus (ranging from out of control to in control). From a different theoretical point of view, discrete-emotion proposals assume a limited number of innate and universal emotional categories, which mainly include happiness, anger, fear, sadness and disgust [3, 4, 5].

Developments in the field of the interplay between emotion and language would not have been possible without the publication of several normative databases in different languages. These norming studies have mainly provided ratings for the affective dimensions of valence and arousal. In this sense, the Affective Norms for English words (ANEW) [1] reports scores for 1034 words in the dimensions of valence, arousal and dominance. This corpus has been adapted to multiple languages including Italian [6], Spanish [7] or European Portuguese [8]. Besides, valence and arousal norms for different sets of words have been reported in several studies (German: [9, 10]; English: [11, 12]; French: [13]; Dutch: [14, 15, 16]; Finnish: [17, 18]; Polish: [19]; Spanish [20, 21, 22, 23, 24, 25]). By contrast, normative studies collecting word ratings for discrete emotional categories are rather scarce (English: [26, 27]; German: [28]; Spanish [24; 29]).

The majority of the aforementioned studies provided ratings not only for affective variables but also for several non-emotional psycholinguistic. Besides reporting objective measures based on corpus statistics -such as word frequency, word length or orthographic neighborhood-, ratings for several subjective variables including concreteness [14, 6], imageability [30, 15], familiarity [31, 22, 7] or subjective age of acquisition (AoA) [11, 16] have been collected. Based on these data, it has been possible to outline some conclusions regarding the interplay between emotion and language at both the word and sentence levels. In this sense, the results of several studies have revealed the involvement of lexico-sematic, phonological or morphosyntactic informationon the processing of emotional language [32, 33, 34, 35, 36, 37, 38, 39, 40, 41, 42, 43, 44, 45]. However, there are many other questions that remain less understood, such as the effects of subjective AoA in the processing of affective language.

The goal of the current study was twofold. As a first objective we aimed to collect ratings for additional subjective affective and psycholinguistic variables for the 875 emotional words included in the Madrid Affective Dataset (MADs, [24]), which currently includes norms for the affective dimensions of valence and arousal, as well as for the discrete dimensions of happiness, fear, anger, sadness and disgust and the lexical variable of concreteness. Scores on new affective and lexicosemantic dimensions may be a useful tool to for designing experiments that address unanswered theoretical and methodological questions on the interplay between language and emotion. In particular, ratings for the following variables were collected in the current normative study:

*●Dominance*. As previously noted, dominance can be defined in terms of feelings of control over an event as opposed to a feeling of being controlled by a situation. Compared to the dimensions of valence and arousal, the dominance dimension is characterized by specific vocal responses and action tendencies, such as wanting to take initiative versus being apathetic [46]. Nonetheless, to the best of our knowledge the role of dominance on the interactions between language and emotion has been not considered.

*●Familiarity*. Familiarity may be defined as the experience that an individual has with a given word [47]. Although familiarity may be related to word frequency, it has been found to be a better predictor of verbal performance than frequency [48]. Also, dissociable contributions of frequency and familiarity have been reported in lexical decision and naming tasks [49; 50]. Finally, it has been shown that words showing a similar frequency varied widely in familiarity, and vice versa [51; 52]. Interestingly, prior studies have established the existence of a relationship between affective dimensions and familiarity [6, 22, 12]. Thus, it may be relevant to consider this variable when designing experiments addressing the processing of affective language.

*●Subjective AoA*, This psycholinguistic variable represents the average chronological age at which a particular word is first learned. Although this measure is based on adult estimates of when different words were learned, the results of several studies have shown that rated AoA and objective AoA -based on children’s naming performance- show a comparable predictive power for picture naming [53]; but see[54]. Also, AoA effects on word comprehension and production tasks have been shown to differ from those elicited by word frequency or familiarity [55,56, 57,58];see [59] for review]. Importantly, several studies have reported specific effects of subjective AoA during the access to orthographic, phonological and semantic representations in non-emotional language [60, 61]. Thus, having available current ratings may be suitable for directly addressing the influence of the age at which words were learned on affective language processing.

*●Sensory experience ratings (SERs)*. Sensory experience is a recently developed concept that refers to “the extent to which a word evokes a sensory and/or perceptual experience in the mind of the reader” ([62], p. 160). Although it may be closely related to imageability, it has been suggested that imageability is biased towards visual sensory activation, whereas SERs captures this information along with other sensory modalities [62, 63]. Interestingly, this variable is a reliable predictor of lexical decision response times [64]. So far, there are currently no available data for this variable for affective words. Interestingly, these norms may be of potential interest for researches investigating affective language under the grounded/embodied cognition perspective of semantic representation, which assumes that conceptual processing is rooted in the individual’s perceptual and motor experiences [65, 66, 67, 68, 69, 70]).

A second goal of this study was to explore the relations between the norms for dominance collected here and the scores for both affective dimensions and discrete emotions that are currently available in the MADs through regression and correlational analyses. Prior work generally showed that people associates feelings of power to highly pleasant and unpleasant words [6, 16, 10]. Also, the relation between dominance and discrete emotions was investigated in a previous study [26], which found that words with higher ratings in happiness, anger, sadness, fear and disgust also scored higher in the dominance dimension.

Additionally, we investigated if the emotional impact of a word is likely to be modulated by other lexicosemantic features. For this purpose we examined the relation between the ratings on the affective variables of the MADs- those previously available and the one currently collected- and these collected in the present study for the psycholinguistic variables of familiarity, subjective AoA and SERs. To the best of our knowledge the relationship between discrete emotions and either familiarity, subjective AoA or SERs remains unexplored. In contrast, prior work has shown a relation between familiarity and both dominance and valence. In this sense, whereas people felt more dominant and happier with concepts denoted by words which they are familiar [6, 22, 11, 12], unfamiliar words tend to be perceived as being more exciting [12];but see [11]. Modulations in the use of emotional language by the AoA of the words have been also described. In this sense, children start using concepts in a broad sense–including all emotions of the same valence-, and then gradually narrow to the use of discrete emotions over a relatively long period of years [71, 72]. Also, words estimated to have been learned at early stages have found to be mainly pleasant, calm and dominant in previous research [11, 12]. Finally, although the relation between SERs and affective dimensions has deserved little attention, previous evidence indicates a positive correlation between valence and some sensory features, such as vivid colors or pleasant taste. Also, high arousal ratings have been associated with words denoting unpleasant taste [12]. Thus, we expected that the results of the correlational analyses reveal the existence of a complex and independent pattern of relations between different lexicosemantic and emotional variables, which make guide future empirical studies on the interplay between language and emotion.

## Materials and Methods

### Participants

Data from 540 native Spanish participants (398 females, 142 males; mean age = 24.31 years, *S*.*D*. = 9.03) were collected. Most of them were students from two universities in Madrid (Universidad Complutense and Universidad Nacional de Educación a Distancia, UNED), but several nonstudent participants took part in the normative study as well. They participated in the study either voluntarily or in exchange for extra course credits. Ratings were collected between February 2014 and September 2014). The study was conducted in accordance with the Declaration of Helsinki and approved by the ethics committee of the Instituto Pluridisciplinar, UCM, Madrid, Spain. Written informed consent was obtained from all participants.

### Materials and procedure

This study used the word-set that was previously selected for [24]. The total 875 Spanish words were randomly distributed into 9 lists of 97(±1) words. Two different types of questionnaires per list were created using the SurveyMonkey Web software, making it a total of 18 questionnaires. The links to the online surveys were randomly distributed among our sample, so that 30 participants rated each one of them.

One of the versions of the questionnaires collected ratings for familiarity and dominance and was completed by half of the participants. The other version collected the subjective AoA and SERs from the remaining half of the participants. Both of them began with a page that contained some initial demographic questions (age and sex) and explained the nature of the study, which included an estimate of completion time (20–25 minutes). Data confidentiality was also addressed here and an e-mail contact was included, in case any of the participants wanted to enquire about our research. Finally, they were provided with the instructions for completion of the ratings for each variable, which were adapted from [1] for dominance, [22] for familiarity, [73] for subjective AoA and [64] for sensory experience, respectively. The instructions that were used can be found in the Appendix.

After the initial page, the questionnaires were designed so that each word would be presented alone in each page, in the center of the screen, in black 14-point Helvetica bold font. Below the stimulus words were the two relevant variables to rate. In the familiarity and dominance questionnaire, both dimensions had a 9-point scale and familiarity was fixed as the first variable to rate, in order to prevent that participants felt more familiar with the concepts if words were previously rated for dominance. In the case of the scale of dominance, the instructions included a pictorial depiction of the Self-assessment Manikin (SAM). This is a widely used tool in the assessment of the emotional attributes of stimuli. The dominance scale shows SAM ranging from a very small to a very large figure representing a feeling of being either submissive or powerful, respectively [74]. In the subjective AoA and SERs version, subjective AoA was fixed as the first variable to rate, using an 11-point scale in which 1 meant “less than 2 years old”, numbers 2–10 indicated the ages 2–10, and a score of 11 meant “11 years or older” [73]. For the SERs variable, a 7-point scale was chosen [63, 47], with the greater numbers indicating a larger degree of sensory experience. All of the items included a response option labeled “I don’t know the meaning” (mean responses per word = 0.25, *SD* = 1.09).

The order of presentation of the words was randomized for each participant in all of the questionnaires. It was also set that once the ratings for a word were submitted, participants were not allowed to go back and change them. Labels were provided to the extreme values in each of the scales, and to all of the values in the subjective AoA scale, to remind the raters of the direction of the polarity of the dimensions.

## Results and Discussion

### The data base

The descriptive statistics for dominance, familiarity, subjective AoA and SERs are summarized in Table 1. This corpus can be accessed as supplementary material from https://figshare.com/s/8e7b445b729527262c88.

**Table 1:** Descriptive stimulus characteristics.

	Dominance	Familiarity	Subjective AoA	SERs
Mean	5.13	5.78	7.07	3.80
*SD*	1.20	1.16	2.16	0.85
Minimum	2.27	2.24	1.53	1.48
Maximum	8.33	8.60	10.88	6.20
Range	6.06	6.36	9.35	4.72

#### Reliability and validity

The reliability of the values for dominance, familiarity, subjective AoA and sensory experience was determined according to the split-half intergroup procedure. Participants were randomly divided into two subgroups of equal size for each version of the questionnaires. Thereafter, we calculated the Pearson correlation coefficients between participant’s scores for the four variables. The adjusted correlations using the Spearman–Brown formula were very high for all the variables. For the dominance dimension, the mean correlation value was *r* = .89 (ranging from *r* = .76 to .94). The split-half reliability for dominance was similar to that found for the arousal dimension in our prior study (*r* = .89) and lower compared to the correlation observed for the valence dimension (*r* = .94). These findings agree with the results of prior studies [6, 16, 12] and indicate that ratings for arousal and dominance presented more variability than those collected for the valence dimension. High correlations were also observed for subjective AoA (mean *r* = .98, ranging from *r* = .97 to .99), familiarity (mean *r* = .94, ranging from *r* = .93 to .96) and SERs (mean *r* = .90, ranging from *r* = .85 to .96), which are in line with the results of previous normative studies [22, 11, 16, 12, 23, 63].

With regard to validity, we compared subjective AoA and familiarity values for some of the words included in our database that have already been rated in other normative studies. Sensory experience values have not been previously collected in Spanish, so we were not able to compare our scores with any other sources. We also could not conduct any validity analysis for the dominance dimension, since ratings for this dimension are currently available for only 16 of the words included in the MADs [7]. The number of words that have been not previously scored in Spanish was 859 for dominance, 459 for familiarity and 240 for subjective AoA. It should be noted that ratings which were already available came from different corpora, so providing scores collected in a homogenous way for the four variables and incorporating them to the same database may be advantageous [23]. For our subjective AoA scores we observed high positive correlations with the ratings of [73], *r*(417) = .95, *p* < .001, and [23], *r*(294) = .95, *p* < .001). The databases by [75] and [76] reported subjective AoA for Spanish words. However, they only included 26 and 0 words, respectively, which were also scored in our corpus. Thus, no reliable results regarding validity could be derived from a direct comparison between scores in these normative studies and those provided here.

Also, familiarity ratings were compared with those reported in the study of [23], *r*(134) = .77, *p* < .001), as well as with those found in the Espal [77], *r*(404) = .59, *p* < .001). Again, even though ratings for familiarity for 8 words that were also included in our normative study were provided in [22], the small number of words shared by the tow corpora does not allow to reliably estimating validity. Altogether, the results of these analyses suggest that our data show a high degree of consistency with ratings for the same variables collected in prior normative studies, even though some methodological differences exist among them.

### Relations among word features

#### Relations between dominance and affective variables

Relative to valence and arousal, the dominance dimension has received little attention in prior research since it accounts for a much smaller proportion of the variance of emotion. Thus, it is not surprising that only few studies have investigated the relation between dominance and the two other affective dimensions. Prior research has typically observed a positive correlation between dominance and valence, with people feeling more in control of words denoting pleasant concepts [6, 16, 8, 10, 12]. Results are more inconsistent with regard to the dimension of arousal. In this sense, some studies found that highly activating words were associated with high scores in dominance [10], whereas others found the opposite pattern of results [12], or even a U-shape relation between dominance and arousal [6, 12]. The results of these studies have some theoretical implications for the debate concerning the orthogonality of the affective dimensions, as it was first established by some authors e.g.,[1]. In order to add some light to this debate, the relation between dominance values and the ratings for both valence and arousal dimensions collected in our prior study [24] was explored in two separate regression analyses, with either valence or arousal as the independent measure and dominance as the dependent one. The linear and quadratic models were tested separately.

**Fig 1:**
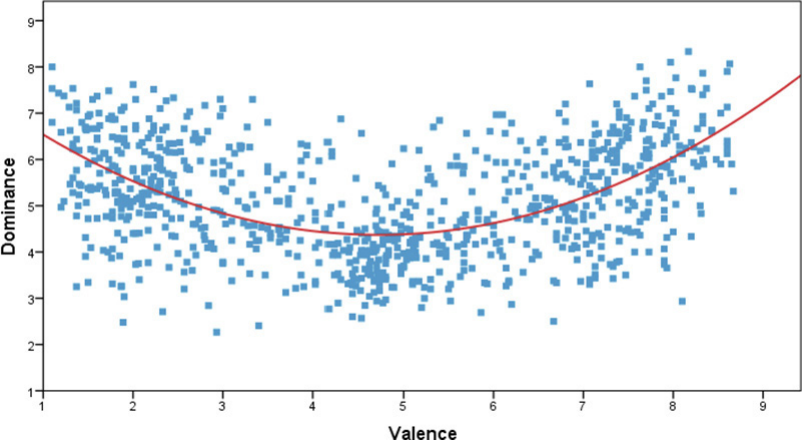
Quadratic distribution of scores for the 875 words in the affective space defined by dominance and valence, for the total sample.

**Fig 2:**
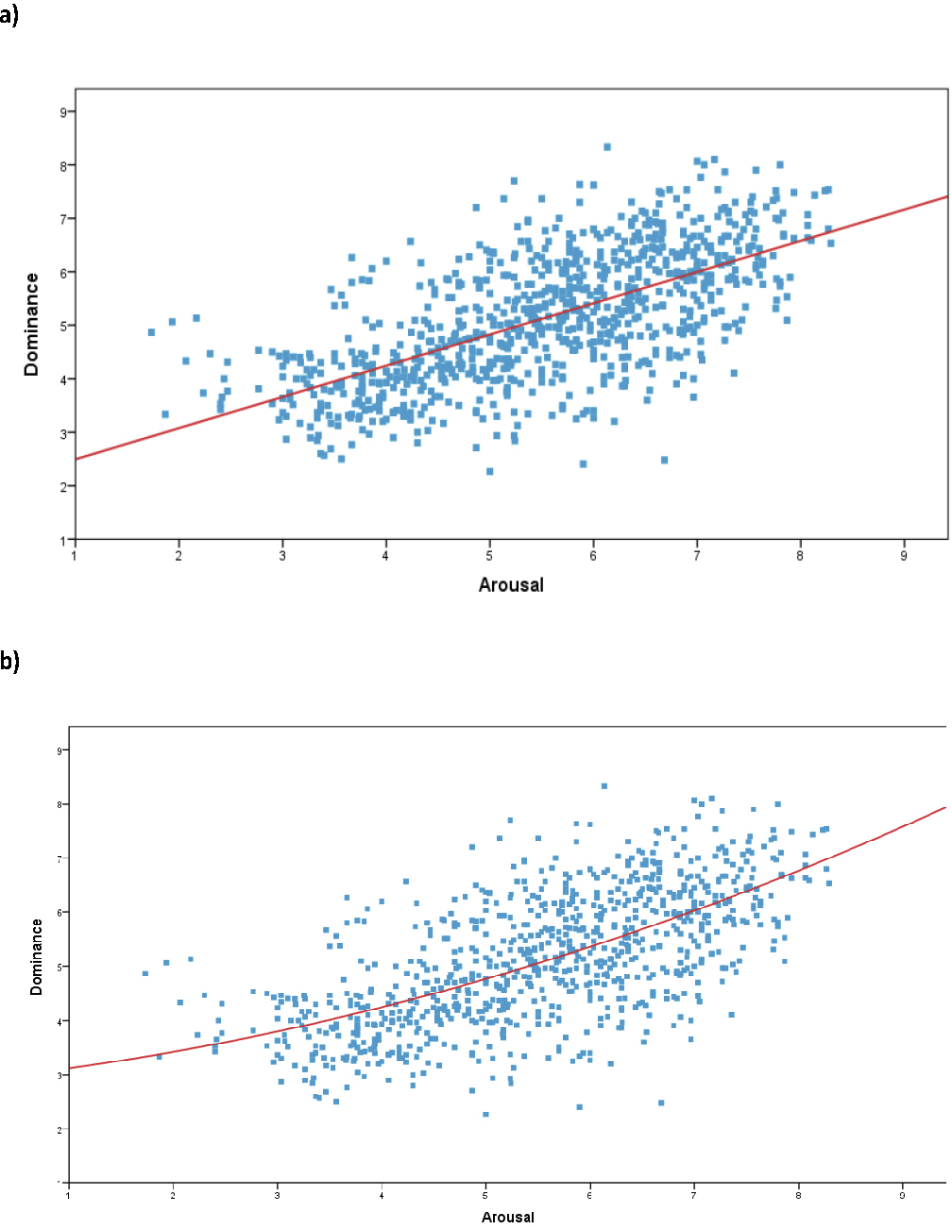
**a) Linear and b) quadratic distributions of scores for the 875 words in the affective space defined by dominance and arousal, for the total sample**.

The affective space defined by the valence and dominance dimensions is shown in Fig 1. A high quadratic relationship between valence and dominance was found, *R* = .52, *F*(2,872) = 162.36, *p* < .001, which explained 27,1% of the variance. The analysis of the linear model showed no significant effects, *R* = .03, *F*(1,873) = 0.84, *p* > .05, accounting only for 0.01% of the variance. The U-shape distribution indicates that participants experienced a higher degree of control to concepts denoted by highly pleasant and unpleasant words. Further analyses confirmed this finding by showing a positive correlation between dominance and valence in pleasant words (mean valence ratings > 6; *r* = .39, *p* < .001) and a negative correlation between dominance and valence in negative words negative words (mean valence ratings < 4; *r* = -.29, *p* < .001). With regard to the relation between dominance and arousal, both the linear [*R* = .65, *F*(1,873) = 632.39, *p* < .001] and the quadratic [*R* = .65, *F*(2,872) = 320.70, *p* < .001] models showed significant effects, accounting for a similar percentage of variance (42% and 42,4%, respectively). Fig 2 shows the affective space defined by the valence and dominance dimensions. Additional analyses showed a positive correlation between dominance and arousal high arousal words (mean arousal ratings > 6; *r* = .37, *p* < .001). In contrast, the correlation between dominance and arousal in low arousal words failed to reach significance (mean arousal ratings < 4; *r* = .004, *p* > .05). Thus, it seems that words associated with higher levels of activation were also experienced as being more under the control of the participants. Finally, the relation between scores for dominance and those collected in our previous normative study [24] for the discrete categories of happiness, fear, anger, sadness and disgust was investigated. We observed a positive correlation between dominance and all discrete emotions, with the highest values for words associated to anger and fear concepts (see Table 2).

**Table 2:** Correlations between dominance (Dom), Familiarity (Fam), subjective age of acquisition (SAoA) and sensory experience ratings (SERs) and the affective variables of valence (Val), arousal (Aro) and the discrete emotions (happiness, Hap; anger, Ang; sadness, Sad; fear, Fea; disgust, Dis).

	Val	Aro	Dom	Hap	Ang	Sad	Fea	Dis
Dom	.031	.648*	1	.209*	.310*	.194*	.362*	.154*
Fam	.399*	.027	.236*	.426*	-.244*	-.232*	-.241*	-.271*
SAoA	-.270*	.019	.006	-.279*	.231*	.214*	.173*	.201*
SERs	.012	.525*	.490*	.235*	.239*	.300*	.339*	.232*

* *p* < .001

Overall, the results of the analyses showed a complex pattern of relationships between dominance and the affective variables. The finding of a quadratic relation between valence and dominance contrasts with prior research that reported a linear relation between these dimensions [6, 12] that is, high dominance in positive and low dominance in negative situations. Several factors may account for this discrepancy. It should be noted first that the correlations reported in a normative study reflect the structure of the specific words used [16]. In this sense, most of the words in our database were rated by the participants as having rather medium scores in the dominance dimension (464 out of 875 words showed values between 4 and 6; 236 words were rated higher than 6; 175 words showed scores lower than 4), which may have undermined the relation between valence and dominance. Moreover, as has been pointed out by some authors [16] differences in rating instructions may influence dominance values. For instance, participants in the current study were instructed to score the dominant meaning of the words, whereas in other studies they rated their own feeling of control (e.g., [12]). Thus, following the argument of [16], a participant may consider a spider as having dominant meaning but feeling a low level of control. Also, the existence of cultural differences in the degree of control that people perceive over affective experiences may not be totally disregarded. In this respect, dominance ratings show greater variability across languages [6, 7]. Also, it has been suggested that dominance scores show a higher sensibility to individual differences since this dimension highlights the raters’ subjective self-centered perspective and participants rely more on their own coping strategies towards an object [10, 74]. Finally, it has been shown that discrete specific that share a negative connotation may be related to very different feelings of power or weakness [46]. In this sense, emotions such as anger have been found to be opposed to sadness or shame in the dominance dimension. Thus, divergent results in dominance ratings across normative studies could be partially attributed to differences in the percentage of negative words belonging to these specific emotions. Nonetheless, the finding of a positive correlation between arousal and dominance replicates the results from previous studies [6, 16, 10]. Also, our data matches those of the only prior study that assessed the relation between dominance and discrete emotions in English words by showing that words rated with higher scores in happiness, anger, sadness, fear and disgust are experienced as being more dominant [26].

To summarize, it seems that results regarding the relation between dominance and other affective variables should be taken with caution given the concerns mentioned above (see [8] for extended discussion on this issue). Here, we provided evidence suggesting that the relation between dominance and valence in a set of Spanish words differs from that observed in Italian [6] Dutch [16] or English [12]. Thus, further corpora are needed in different languages and including different sets of words in order to clarify this issue. At a theoretical level, the finding of a significant relation between dominance and discrete emotions suggests that it is important to design studies that consider the scores in affective categories and dimensions in order to further develop the claims made by both dimensional and basic emotion proposals [24, 26]).

#### Relations between psycholinguistics and affective variables

We also explored the influence of ratings collected for familiarity, subjective AoA and sensory experience on the assessments of the affective content of words. To this end, Pearson correlation coefficients were computed between these psycholinguistic variables and dominance scores found in the current study. Ratings on psycholinguistics variables were also correlated with those collected in our previous study for the values of the affective dimensions of valence and arousal, as well as for the emotional categories of happiness, anger, sadness, fear and disgust. The results of these analyses are summarized in Table 2.

Familiarity values showed significant positive correlations with scores in valence, suggesting that more familiar words also tend to denote positive concepts. We also found a positive correlation between familiarity and dominance. Thus, participants felt more control when they assessed words that occur more often in everyday language. In contrast, no significant correlation was found for familiarity and arousal. Although familiarity is thought to be more language specific than other lexical variables [17], the relation between familiarity and affective dimensions closely replicates the results from previous studies [6, 22, 12]. These findings have been interpreted to reflect an adaptive tendency to feel threatened and to perceive lower control when dealing with something unfamiliar [6]. In agreement with this view, we observed a positive significant correlation between familiarity and happiness while negative significant correlations were found with anger, sadness, fear and disgust.

Subjective AoA had a negative correlation with valence, whereas no significant correlations were found with either arousal or dominance. This finding suggests that words rated as denoting more pleasant concepts are learned early in life. Similar findings have been reported by [16], although a positive correlation between subjective AoA and dominance was also found in their study. In contrast, [11] only found a relation between subjective AoA and arousal. Again, divergences could be explained by differences in language characteristics, stimulus sets and sample peculiarities. Notably, the relation between subjective AoA and affective dimensions has been the subject of just the two above mentioned studies, so future research will provide additional clues on this issue. Regarding discrete emotions, subjective AoA showed a negative correlation with happiness, while positive correlations were observed for angry, sad, fearful and disgusting words. Thus, our data indicate a learning advantage for happy words, whereas words related to negative discrete categories are learnt later in life.

Taken together, prior results suggesting the contribution of AoA to word recognition times [33] and those found in the current study highlight the importance of controlling the effects of this variable in studies concerned with the processing of affective language.

Finally, SERs showed a positive correlation with arousal and dominance, which suggests that more activating and dominant words also evoke a richer sensory and perceptual experience. Interestingly, although our results indicate a lack of relation between SERs and valence, positive correlations between SERs and all five discrete emotions were found. Thus, words associated with more vivid sensorial attributes are also those rated with higher scores on happiness, anger, sadness, fear and disgust.

The close relation observed between SERs and both arousal and dominance, on the one hand, and between SERs and emotional categories, on the other hand, may be of particular interest for those researches conducting investigations under the embodied cognition framework. In this sense, our data may be suitable for investigating sensory activation elicited by pleasant and unpleasant words. The current results may also have some theoretical implications for the status of SERs. It should be mentioned that in our prior study we found relations between concreteness and both valence and arousal, as well as with ratings in anger, sadness, fear and disgust. Notably, the divergent pattern of relationships between concreteness or SERs and affective variables, provides indirect evidence for the idea that these psycholinguistic variables may rely on different perceptual attributes, even though both concepts are conceptually related. In this respect, SERs showed a positive correlation with concreteness scores obtained in our prior normative study, *r* = .30, *p* < .001, indicating that words associated with more sensory experiences were also perceived as denoting more concrete concepts. Nonetheless, it has been pointed out that concreteness and imageability tend to stress visual and sound properties, whereas SERs may be associated with a broader set of sensorial modalities, including smells, odors and tastes [62, 63].

Overall, it seems that familiarity, subjective AoA and SERs have distinct relations with the affective properties of the words, which highlight the complex pattern of interactions between different emotional and psycholinguistic features found in prior studies. Our data also emphasizes the importance of taking into account these variables when investigating affective language and formulating theoretical models of emotional language processing.

## Conclusion

The present normative study expands the MADS by incorporating ratings for an affective dimension and three psycholinguistic variables. Thus, researches interested in investigating affective language have now available a corpus of 875 words that includes norms for three affective dimensions–valence, arousal and dominance–, five discrete emotions–happiness, anger, sadness, fear and disgust–, four subjective psycholinguistic variables–concreteness, familiarity, subjective AoA and SERs–, and two objective psycholinguistic variables–word frequency and word length–. Furthermore, the results of our analyses confirmed the relations between affective dimensions and both familiarity and subjective AoA observed in prior studies in other languages. Notably, we reported here for the first time relations between these two psycholinguistic variables and discrete emotional categories, as well as between SERs and emotional variables. In sum, the new data should facilitate the design of empirical experiments that investigate the precise contribution of these psycholinguistic features to the processing of affective language.
